# Smoking cessation and obesity-related morbidities and mortality in a 20-year follow-up study

**DOI:** 10.1371/journal.pone.0279443

**Published:** 2022-12-28

**Authors:** Asla Suutari-Jääskö, Antti Ylitalo, Justiina Ronkaine, Heikki Huikuri, Y. Antero Kesäniemi, Olavi H. Ukkola

**Affiliations:** 1 Research Unit of Internal Medicine, Medical Research Center Oulu, Oulu University Hospital, University of Oulu, Oulu, Finland; 2 Heart Center, Turku University Hospital and University of Turku, Turku, Finland; 3 Center for Life Course Health Research, University of Oulu, Oulu, Finland; North Dakota State University, UNITED STATES

## Abstract

**Background:**

Smoking is the biggest preventable factor causing mortality and morbidity and the health benefits of smoking cessation are commonly known. Smoking cessation-related weight gain is well documented. We evaluated the association between smoking cessation and the incidence of obesity-related morbidities such as hypertension, diabetes and metabolic syndrome as well as mortality. We also evaluated telomere length related to smoking cessation.

**Material and methods:**

This study was part of the OPERA (Oulu Project Elucidating Risk of Atherosclerosis) study. The mean follow up time among the 600 study subjects was 20 years. We divided the study subjects into four groups by smoking status (“never”, “current”, “ex-smokers” and “quit”) and analyzed their health status. “Ex-smokers” had quit smoking before baseline and “quit” quit during the follow-up time. Information about total mortality between the years 2013–2020 was also utilized.

**Results:**

During the follow-up time systolic blood pressure decreased the most in the “current” and in the “ex-smoker” groups. Office SBP decreased the least in the “quit” group (p = 0.001). BMI increased the most in the “quit” and the least in the “ex-smokers” group (p = 0.001). No significant increases were seen in the incidence of obesity-related-diseases, such as metabolic syndrome, hypertension and diabetes was seen. There was no significant difference in the shortening of telomeres. Odds of short-term mortality was increased in the “current” group (2.43 (CI 95% 1.10; 5.39)), but not in the “quit” (1.43 (CI 95% 0.73–2.80)) or “ex-smoker” (1.02 (CI 95% 0.56–1.86)) groups when compared to “never” group.

**Conclusions:**

Even though, the blood pressure levels were unfavorable in the “quit” group, there was no significant increase in the incidence of obesity-related-diseases, and a noticeable benefit in short-term mortality was seen during the 6-year follow-up. The benefits of smoking cessation outweigh the disadvantages in the long-term.

## Introduction

Smoking is the biggest preventable factor causing mortality in the world and therefore smoking cessation is desirable in the general population [1]. The most common mortality-causing diseases related to tobacco smoking are lung cancer, atherosclerotic diseases and chronic obstructive pulmonary disease (COPD) [2]. Smoking cessation has many short- and long-term benefits. For instance, it improves ventilation, enhances the prognosis of COPD and coronary heart disease, and lowers the risk of tobacco-related cancers [3]. Benefits in mortality related to smoking cessation can be noticed in all age groups [2, 4], although the benefit is greatest in young age [3].

Smoking cessation is related to substantial weight gain and increased BMI (body mass index). People who quit smoking gain more weight than those who continue smoking and more than those who have never smoked [5, 6]. The mechanism behind the weight gain is related to decreased metabolic rate, decreased physical activity and eating habits [7]. Mortality and risk of chronic diseases related to smoking seem to decrease over the long term after smoking cessation even though there is substantial weight gain [6, 8–10]. However, some previous studies have reported an increased risk of type 2 diabetes after smoking cessation, partly due to cessation-related weight gain [8, 9], although previous studies have also shown that active smoking is a major risk factor for type 2 diabetes [9].

Hypertension (HTN) is a significant cause of mortality and cardiovascular diseases (CVD) [11], and the combination of tobacco smoking and hypertension increases mortality and the risk of CVD even more [12, 13]. Previous studies have found that current smokers, both men and women, have lower blood pressure levels and lower probability for hypertension diagnosis [11, 13, 14]. Smoking cessation is associated with weight gain compared to those who continue smoking, which leads to increased blood pressure and pulse pressure levels [15, 16]. Some previous studies have reported that smoking cessation increases the risk for hypertension compared to those who continue smoking [11, 16–18]. However, in the long follow-up, smoking appeared to increase the risk for hypertension dose-dependently [19, 20].

Telomeres are protein structures at the end of DNA in eukaryotic cells [21]. During mitosis, in each cell division there is a progressive loss of nucleotides due to DNA polymerase enzymes’ inability to duplicate the lagging strand, which leads to telomere shortening. Eventually, the telomere shortening leads to cellular aging and apoptosis [22]. Several studies have reported that tobacco smoking accelerates the shortening of telomeres dose-dependently [21, 23–26]. The shortening is due to increased oxidative stress and chronic systemic inflammation caused by tobacco exposure [21, 27]. Telomeres also shorten steadily with age [24] and females have relatively longer telomeres than males [23]. In this study, with such a long follow-up time, we wanted to evaluate how much smoking accelerates telomere shortening over time. We also wanted to examine whether smoking cessation would have a positive effect on the accelerated shortening of the telomeres.

This research is part of the population-based epidemiological study OPERA (Oulu Project Elucidating Risk of Atherosclerosis) which evaluates the health of the study population very extensively. However, the greatest benefit of this study is its relatively long, 20-year follow-up. We divided the study subjects into four groups by their smoking status (never smokers “never”, current smokers “current”, those who had quit smoking before follow-up time “ex-smokers”, and to those who quit during follow-up “quit”). This study did not consider any possible relapses after smoking cessation. Our aim was to examine how tobacco exposure and smoking cessation affected their health in the long term. We monitored the groups for incidence of diabetes, HTN, usage of antihypertensive drugs and obesity. Additionally, we examined how tobacco smoking affected the subjects’ telomere length. Our main purpose was to examine how smoking and smoking cessation affected the incidence of diseases and total mortality during the 6 years following the cessation. This 6-year examination was performed by utilizing the information of total mortality of the study subjects between the years 2013–2020.

## Subjects and methods

OPERA (Oulu Project Elucidating Risk of Atherosclerosis) is a population-based epidemiological study focusing on the risk factors and disease end points of atherosclerotic cardiovascular diseases. In the first phase, OPERA was a cross-sectional study of middle-aged (40–59 years) subjects (n = 1,045) with hypertension (n = 519) and their age- and sex-matched controls (n = 526) [28, 29]. The first phase was conducted between 1991 and 1993. In the second phase (2013–2014), study subjects were recruited to a follow-up visit. A total of 600 subjects attended the visit; at the time, the study subjects were 63–83 years old. Examinations performed both at baseline and follow-up included weight, height, waist and hip measurements, BMI as well as blood pressure measurements. A questionnaire presented to all participants provided information on their smoking habits, alcohol consumption, physical activity, use of medication and past medical history. Alcohol consumption was calculated as grams of absolute alcohol consumed per week and smoking as the number of cigarettes smoked per day [30]. The life-time smoking burden was calculated as pack-years (1 pack-year = 20 cigarettes smoked/day in one year) and the smoking history was obtained from a questionnaire [29].

We used the IDF (International Diabetes Federation) definition of metabolic syndrome, and insulin sensitivity was measured using the QUICK index (quantitative insulin sensitivity check index) as described earlier [30]. Hypertension was defined as BP (blood pressure) above 140/90 mmHg or current antihypertensive medication [30]. Ambulatory blood pressure measurements were obtained with a non-invasive fully automatic SpaceLabs90207 oscillometric unit (SpaceLabs Inc., Redmond, WA). The measurements were taken every 15 min from 04:00 am to 12:00 pm and every 20 min from 12:00 pm to 04:00 am [29].

The routine clinical laboratory tests at the baseline visit were carried out in the Central Laboratory of Oulu University Hospital after 12-h fasting [28]. The methods concerning measurement of fasting total ghrelin, leptin, adiponectin and resistin [29, 31, 32] have been described earlier. The routine laboratory tests during the follow-up period were carried out in the Joint Municipal Service Provider of Northern Finland Laboratory Center, NordLab Oulu (since 2013; prior to that, Laboratory of the Oulu University Hospital) [30]. Plasma ghrelin was measured with the Merck Millipore ELISA (EZGRT-89K) method.

To study telomeres, we used relative leukocyte telomere length. This was determined by measuring the PCR amplification of the telomeric DNA sequence (T) and comparing it to that of a single copy gene (S) using a T/S ratio for each study subject. Genomic DNA of leukocyte telomeres was measured from peripheral blood samples taken at the baseline and at follow-up using a multiplex quantitative real-time PCR method [33] with minor modifications as described in Korkiakoski A et al. 2021 study [34]. Briefly, T/S ratio was assessed using albumin as single copy gene by Albugcr2 and Albdgcr2 primers. The mean R2 values for standard curves were 0.991 and 0.995 for T and S, respectively and the mean coefficient of variation for all T/S values was 5.31%.

### Outcome classification

After the follow-up examinations and laboratory tests (which were conducted between 2013 and 2014), the participants were followed up for an average 77 months, the end of this short follow-up being death or the last day of 2020. Information on total mortality was obtained from the Finnish Causes of Death Register.

### Statistical methods

Data was analyzed using IBM SPSS Statistics version 27. We divided the study population into the four groups by smoking status. Never smokers formed the first group (“never”). People who smoked after the follow-up time formed the second group (“current”). Those who had quit smoking before the baseline formed the third group (“ex-smokers”) and those who quit during the follow-up formed the fourth group (“quit”). In addition to pooled analyses blood pressure levels, telomere length, alcohol consumption and smoking pack years were also analyzed separately by sex. The analysis of variance (ANOVA) was used to compare more than two groups with continuous variables. Post hoc analyses for differences between groups for differences were conducted with Tukey´s statistical test. Chi-square-test (χ2) was used with categorical variables. P-values under 0.05 were considered statistically significant.

The effect of the confounding factors with the main results was noticed and tested with univariate model. As covariates we used age, sex, baseline values and BMI change. The association between smoking status groups and total mortality during the follow-up time was estimated with Cox proportional hazards models.

### Ethical considerations

The study was approved by the Ethics Committee of the Medical Department of the University of Oulu (48/2009). Written informed consent was given by the participants for the use of their clinical records to be used in this study.

## Results

Smoking status groups consisted of never smokers (n = 306) (“never”), smokers (n = 45) (“current”) and those who had quit smoking before baseline (“ex-smoker”) (n = 151) or during the follow-up (“quit”) (n = 98) of the study.

### Baseline examination

Females were overrepresented in the “never” group and underrepresented in the “quit”-group (p < 0.001) (Table 1). “Current” subjects were the youngest at the baseline examination (p = 0.002). The “ex-smoker” group had the highest BMI at baseline (p = 0.008). At baseline there were no significant differences between the groups in the incidences of HTN, usage of antihypertensive drugs, diabetes or metabolic syndrome.

**Table 1:** Study subjects’ clinical characteristics according to smoking status.

	Never	Current	Ex- smokers	Quit	p-value	post hoc	p after adjustments
	n = 306	n = 45	n = 151	n = 98			
**Age (yrs)**	50.6	47.5	50.2	49.2	0.002	a**, d*	
**Female N (%)**	211 (69.0)	20 (44.4)	42 (27.8)	46 (46.9)	0.000		
**Metabolic syndrome n (%)**							
**At the baseline**	92 (30.1)	13 (28.9)	61 (40.4)	30 (30.6)	0.137		
**At the follow-up**	187 (61.1)	28 (62.2)	97 (64.2)	60 (61.2)	0.930		
**Hypertension n (%)**							
**At the baseline**	153 (50.0)	23 (42.2)	76 (55.0)	49 (41.8)	0.138		
**At the follow-up**	222 (72.5)	37 (82.2)	116 (76.8)	75 (76.5)	0.454		
**Antihypertensive drug n (%)**							
**At the baseline**	152 (49.7)	18 (40.0)	81 (53.6)	48 (49.0)	0.449		
**At the follow-up**	230 (75.2)	39 (86.7)	118 (78.1)	79 (80.6)	0.294		
**Diabetes n (%)**							
**At the baseline**	20 (7.8)	3 (6.7)	10 (7.3)	7 (2.0)	0.246		
**At the follow-up**	100 (32.7)	18 (40.0)	61 (40.4)	37 (37.8)	0.365		
**BMI (kg/m2)**							
**At the baseline**	26.9	27.2	28.4	27.5	0.008	b**	0.042
**At the follow-up**	28.6	28.6	29.5	30.4	0.013		
**Change**	1.7	1.4	1.1	2.9	0.001	c*, f***	0.002
**Height (cm)**							
**At the baseline**	165.8	170.4	172.4	167.9	0.000	a, f**, b***, c*	0.035
**At the follow-up**	163.1	167.1	169.7	164.8	0.000		
**Change**	-2.7	-3.3	-2.7	-3.1	0.040		0.002
**Weight (kg)**							
**At the baseline**	74.1	78.9	84.8	78.0	0.000	b***, f**	0.007
**At the follow-up**	76.1	79.5	85.1	82.9	0.000		
**Change**	2.0	0.6	0.3	4.9	0.002	f**	0.011
**Waist (cm)**							
**At the baseline**	86.0	91.3	95.1	90.1	0.000	a, c*, f**, b***	0.009
**At the follow-up**	93.7	99.1	100.8	100.6	0.000		
**Change**	7.7	7.8	5.7	10.5	0.001	f**	0.005
**Alcohol consumption (g/wk)**							
**At the baseline**	33.1	85.3	67.4	70.6	0.000	a, b, c***	0.000
**At the follow-up**	27.4	60.0	49.8	50.4	0.000		
**Change**	-5.7	-25.3	-17.6	-20.2	0.074		0.917
**Smoking pack- years**							
**At the baseline**	0.2	18.7	12.2	18.0	0.000	a, b, c, d, f***	0.000
**At the follow-up**	0.3	30.5	13.6	28.0	0.000		
**Change**	0.1	11.8	1.4	10.0	0.001	a, d, f***, c, e**	0.000

BMI, body mass index. Data for continuous variables tested with ANOVA. Categorical variables as prevalences (%) and tested with Pearson Chi-square test. P-values under 0.05 were considered statistically significant. For post hoc analyses

*P<0.05

**P<0.01

***P<0.001, analyzed using ANOVA Tukey statistical test (95% confidence interval) for differences between groups a) 1 vs 2; b) 1 vs 3 c) 1 vs 4; d) 2 vs 3; e) 2 vs 4; f) 3 vs 4.

At baseline examination, ex-smokers had the highest office systolic blood pressure (SBP) (p = 0.029) and pulse pressure (PP) (p = 0.037) (Table 2). In baseline ambulatory measurements, the “current” group had the highest 24h, day- and nighttime DBP (diastolic blood pressure) (p-values 0.024, 0.049 and 0.031, respectively). The highest heart rate (HR) levels in office (p = 0.047) and in 24h and day monitoring (p = 0.001 and <0.001, respectively) were also seen in the “current” group.

**Table 2:** Study subjects’ blood pressure, pulse pressure and heart rate values according to smoking status.

	Never	Current	Ex-smokers	Quit	p-value	post hoc	p after adjustments
	n = 306	n = 45	n = 151	n = 98			
**SBP office**							
**At the baseline**	145	143	149	141	0.029	f*	0.079
**At the follow-up**	140	130	136	140	0.016		
**Change**	-5	-13	-13	-1	0.001	b*, f**	0.014
**SBP 24h monitoring**							
**At the baseline**	128	132	129	128	0.316		
**At the follow-up**	133	130	133	135	0.270		
**Change**	5	-2	4	8	0.050	e*	0.205
**SBP day monitoring**							
**At the baseline**	132	137	134	133	0.196		
**At the follow-up**	134	132	134	137	0.337		
**Change**	2	-5	0	4	0.060	e*	0.108
**SBP night monitoring**							
**At the baseline**	115	117	117	114	0.245		
**At the follow-up**	124	119	126	126	0.185		
**Change**	9	2	9	13	0.090		
**DBP office**							
**At the baseline**	87	87	90	87	0.076		
**At the follow-up**	72	69	73	74	0.064		
**Change**	-15	-18	-17	-13	0.049		
**DBP 24h monitoring**							
**At the baseline**	80	84	82	80	0.024	a*	0.100
**At the follow-up**	72	72	73	74	0.074		
**Change**	-8	-12	-9	-6	0.014	e**	0.188
**DBP day monitoring**							
**At the baseline**	84	88	85	85	0.049	a*	0.069
**At the follow-up**	73	73	74	76	0.144		
**Change**	-11	-15	-11	-9	0.045	e*	0.337
**DBP night monitoring**							
**At the baseline**	69	72	72	69	0.031		
**At the follow-up**	64	63	67	68	0.005		
**Change**	-5	-9	-5	-1	0.019	e*	0.125
**HR office**							
**At the baseline**	73	76	72	70	0.047		
**At the follow-up**	69	66	70	69	0.271		
**Change**	-4	-10	-2	-1	0.004	d, e*	0.117
**HR 24h monitoring**							
**At the baseline**	69	75	68	70	0.001	a**, d***	0.000
**At the follow-up**	65	67	65	66	0.315		
**Change**	-4	-8	-2	-5	0.012	d**	0.257
**HR day monitoring**							
**At the baseline**	72	79	71	73	0.000	a**, d***	0.000
**At the follow-up**	66	68	66	67	0.325		
**Change**	-6	-11	-4	-7	0.003	a*, d**	0.298
**HR night monitoring**							
**At the baseline**	61	64	61	62	0.071		
**At the follow-up**	60	62	61	62	0.170		
**Change**	-1	-3	1	-1	0.066		
**PP office**							
**At the baseline**	58.3	57.3	58.5	53.6	0.037	c*	0.078
**At the follow-up**	68.2	61.9	63.3	65.8	0.019		
**Change**	9.8	4.4	4.8	12.2	0.007	f*	0.171
**PP 24h monitoring**							
**At the baseline**	48.2	48.0	47.5	47.4	0.796		
**At the follow-up**	60.8	58.7	59.9	61.1	0.671		
**Change**	13.0	10.8	12.8	14.2	0.485		
**PP day monitoring**							
**At the baseline**	48.6	49.0	48.3	48.1	0.936		
**At the follow-up**	61.0	58.8	60.2	61.4	0.660		
**Change**	12.8	9.8	12.3	13.8	0.378		
**PP night monitoring**							
**At the baseline**	45.9	45.8	45.4	45.1	0.891		
**At the follow-up**	59.9	56.8	58.8	59.0	0.571		
**Change**	14.5	11.4	14.0	14.4	0.600		

SBP, systolic blood pressure; DBP, diastolic blood pressure; PP, pulse pressure; HR, heart rate. Blood pressure unit mmHg, heart rate unit beats per minute. Data for continuous variables means tested with ANOVA. P-values under 0.05 were considered statistically significant. For post hoc analyses

*P<0.05

**P<0.01

***P<0.001, analyzed using ANOVA Tukey statistical test (95% confidence interval) for differences between groups a) a) 1 vs 2; b) 1 vs 3 c) 1 vs 4; d) 2 vs 3; e) 2 vs 4; f) 3 vs 4.

QUICK insulin sensitivity index (Table 3) was the highest in the “never” and the lowest in the “ex-smoker” group, and the differences were statistically significant between these two groups (p = 0.033). Serum creatinine value was the highest in the “ex-smoker” group at baseline (p = 0.002). In turn, eGFR was the lowest in the “never” group (p < 0.001). Cholesterol and LDL cholesterol levels were the highest in the “ex-smoker” group at baseline (p-values 0.033 and 0.009, respectively). However, the “never” subjects had the highest HDL cholesterol levels (p < 0.001) while those belonging to the “current” group had the highest serum triglyceride concentration (p = 0.003).

**Table 3:** Study subjects’ laboratory values according to smoking status.

	Never	Current	Ex-smokers	Quit	p-value	post hoc	p after adjustments
	n = 306	n = 45	n = 151	n = 98			
**ALAT**							
**At the baseline**	28.3	31.4	32.0	28.4	0.089		
**At the follow-up**	25.3	27.7	27.6	28.6	0.157		
**Change**	-2.9	-3.7	-4.4	0.2	0.228		
**Creatinine**							
**At the baseline**	80.8	76.4	84.5	79.8	0.002	b, f*, e**	0.005
**At the follow-up**	72.3	68.6	80.0	69.5	0.018		
**Change**	-8.5	-7.8	-4.9	-10.3	0.382		0.673
**GFR (CKD-EPI) mL/min**							
**At the baseline**	82.0	93.5	86.4	88.3	0.000	a***, b, e*, d**	0.005
**At the follow-up**	80.2	86.9	81.4	85.0	0.005		
**Change**	-1.8	-6.6	-5.0	-3.3	0.035		0.675
**Quick index**							
**At the baseline**	0.64	0.60	0.60	0.63	0.005	b**	0.265
**At the follow-up**	0.54	0.53	0.53	0.52	0.495		
**Change**	-0.10	-0.07	-0.07	-0.11	0.014	b, f*	0.405
**Fasting glucose**							
**At the baseline**	4.6	4.6	4.6	4.3	0.132		
**At the follow-up**	7.8	7.1	7.2	7.0	0.059		
**Change**	2.0	1.6	2.0	2.3	0.814		
**Fasting insulin**							
**At the baseline**	11.7	12.8	13.9	11.3	0.036	b*	0.314
**At the follow-up**	18.1	17.4	17.2	18.0	0.975		
**Change**	6.4	4.6	3.3	6.7	0.403		0.723
**Cholesterol**							
**At the baseline**	5.5	5.6	5.8	5.6	0.033	b*	0.383
**At the follow-up**	4.8	4.4	4.6	4.8	0.032		
**Change**	-0.7	-1.2	-1.2	-0.8	0.001	b**	0.089
**HDL cholesterol**							
**At the baseline**	1.44	1.26	1.28	1.29	0.000	a*, b***, c**	0.098
**At the follow-up**	1.56	1.34	1.38	1.45	0.000		
**Change**	0.12	0.08	0.10	0.16	0.447		0.363
**LDL cholesterol**							
**At the baseline**	3.35	3.45	3.64	3.54	0.009	b**	0.482
**At the follow-up**	2.85	2.70	2.79	2.93	0.522		
**Change**	-0.50	-0.75	-0.85	-0.61	0.030	b*	0.340
**Triglycerides**							
**At the baseline**	1.35	1.63	1.61	1.57	0.003	b**	0.096
**At the follow-up**	1.25	1.33	1.39	1.39	0.073		
**Change**	-0.10	-0.30	-0.22	-0.18	0.169		0.175
**Hemoglobin**							
**At the baseline**	138.0	144.6	145.1	143.3	0.000	a, c**, b***	0.207
**At the follow-up**	138.0	143.4	143.7	140.0	0.000		
**Change**	0.0	-1.2	-1.3	-3.3	0.228		0.347
**hs-CRP (mg/mL)**							
**At the baseline**	2.672	2.642	4.574	3.345	0.102		
**At the follow-up**	2.731	2.400	2.825	3.558	0.476		
**Change**	0.072	-0.144	-1.747	0.212	0.129		
**Telomere length**							
**At the baseline**	.93	1.07	.99	.98	0.000	a**, b*	0.243
**At the follow-up**	.56	.57	.54	.53	0.299		
**Change**	-0.36	-0.50	-0.45	-0.45	0.000	a, b***, c**	0.896

hs-CRP, high-sensitivity C-reactive protein HDL-cholesterol, high density lipoprotein; LDL-cholesterol, low density lipoprotein; GFR (CKD-EPI), estimated glomerular filtration calculated with CKD-EPI formula (Chronic Kidney Disease Epidemiologic Collaboration). Data for continuous variables tested with ANOVA. P-values under 0.05 were considered statistically significant. For post hoc analyses

*P<0.05

**P<0.01

***P<0.001, analyzed using ANOVA Tukey statistical test (95% confidence interval) for differences between groups a) 1 vs 2; b) 1 vs 3 c) 1 vs 4; d) 2 vs 3; e) 2 vs 4; f) 3 vs 4.

Subjects belonging to the “current” group had the longest telomeres (Table 3) at baseline while those in the “never” group had the shortest (p < 0.001). However, after adjustments for sex and age there was no significant difference between groups.

At baseline the male “current” group consumed the most alcohol (119.6 g/wk, p = 0.024) and “never” the least (66.7g/wk, p = 0.024). Females belonging to the “current” group also consumed the most alcohol (42.4 g/wk, p < 0.001) and “never” the least (18.0 g/wk, p < 0.001).

### Changes during follow-up

#### Weight

BMI changes during the follow-up time differed significantly between the smoking status groups (p = 0.001) (Table 1). BMI increased the most (2.9 kg/m2) in the “quit” group and the least (1.1 kg/m2) among “ex-smokers”. With post hoc analysis, a statistically significant difference was observed between these two groups (p = 0.001). In addition, BMI increased less in the “current” (1.4 kg/m2) than in the “quit” group (p = 0.014). After adjustments for age, sex and baseline BMI, the differences remained statistically significant (p = 0.002). Waist circumference also increased the most in the “quit” subjects (10.5 cm) and the least in “ex-smokers” (5.7 cm), significantly so before (p = 0.001) and after adjustments (p = 0.005) for age, sex and baseline waist circumference. Also the change in the weight was statistically significant after adjustments for sex, age and baseline weight.

Even though there was a noticeable increase in BMI and waist circumference in the “quit” group during the follow-up, there was no significant increase in the incidence of obesity-related diseases such as metabolic syndrome, hypertension and diabetes. The use of antihypertensive drugs did not increase, either.

#### Office blood pressure

When considering the changes in office blood pressure values (Table 2) during the follow-up period, office SBP decreased the most (13 mmHg) in the “current” and in the “ex-smoker” groups. On the other hand, office SBP decreased the least in the “quit” group (1 mmHg p = 0.001). The difference in the office SBP between the “never” and “ex-smoker” groups was significant, as was also the case between the “ex-smoker” and “quit” groups. After adjustments for baseline office SBP, sex and BMI change, the differences in office SBP change between the above-mentioned groups remained statistically significant. Office DBP (diastolic blood pressure) seemed to decrease the most in the “current” group (18 mmHg) and the least in the “quit” group (13 mmHg) (p = 0.049) (Table 2). However there was no statistically significant difference between groups according to post hoc analyse.

We also analyzed males and females separately. Among males, but not females, in the “ex-smoker” group the mean decrease in office SBP was 17.2 mmHg, the same value being 1.8 mmHg (p = 0.002) in the “quit” group. The difference between these two male groups was also statistically significant after adjustments for baseline office SBP and BMI change (p = 0.010).

Office DBP (diastolic blood pressure) decreased the most in the “current” group (18 mmHg) and the least in the “quit” group (13 mmHg) (p = 0.049) (Table 2).

#### Ambulatory blood pressure

During the follow-up, 24-hour SBP and daytime SBP increased the most in the “quit” group (p = 0.050) (Table 2). There was a significant difference in 24-hour SBP and daytime SBP before adjustments between the “current” and the “quit” groups (p-values 0.029 and 0.049): in the “current” group SBP decreased while in the “quit” group it increased. The difference lost its significance after adjustments for baseline SBP, BMI change and sex.

Baseline ambulatory diastolic blood pressure (DBP) (24h, daytime and night-time) was the highest in the “current” group (Table 2) (p-values 0.024, 0.049 and 0.031, respectively) and the lowest in the “never” group. During follow-up, DBP decreased at every monitoring point, the most in the “current” group and the least in the “quit” group (p-values 0.014, 0.045 and 0.019). The difference in DBP was significant between the “current” and the “quit” groups (p-values 0.008 for 24-hour, 0.026 for daytime and p 0.014 for night-time DBP). However, the differences were lost after adjustments for baseline DBP, BMI change and sex.

#### Pulse pressure

During the follow-up time, pulse pressure (Table 2) measured in office conditions (office PP) increased the most in the “quit” group (p = 0.007). On the contrary, a much smaller increase in office PP was seen in the “ex-smoker” and “current” groups. The difference between the “ex-smoker” and “quit” groups was significant (p = 0.021). When adjustments for sex, BMI change and baseline office PP were performed, the differences became non-significant. However, when sexes were considered separately, among the male “ex-smoker” group the mean increase in PP was 1.4 mmHg while in the “quit” group the mean increase was 10.7 mmHg (p = 0.025). The latter difference was also statistically significant after adjustments for baseline PP and BMI change (p = 0.044).

#### Laboratory measurements

During follow-up, changes in cholesterol was the only laboratory value (Table 3) showing statistically significant difference between the groups after adjustments for baseline value, age and BMI. The difference was statistically significant between “never” and “ex-smokers” (p = 0.001). In the “never” group, cholesterol decreased 0.7 mmol/L while in the “ex-smoker” group it decreased 1.2mmol/L. However, the statistically significant difference disappeared after adjusting cholesterol by sex.

We also analyzed obesity-related hormones according to smoking status (Table 4). There seemed to be a visible difference in resistin between “never” and “ex-smokers”, but after adjustments for age, sex, BMI and baseline value the difference was not significant anymore. There were no significant differences between groups in other obesity-related hormones we analyzed.

**Table 4:** Study subjects’ obesity-related hormones according to smoking status.

	Never (1)	Current (2)	Ex-smokers	Quit (4)	p value	post hoc	p after adjustments
	n = 306	n = 45	(3) n = 151	n = 98			
**Leptin**							
**At the baseline**	12.5	9.1	8.4	9.6	0.000	b***, c*	0.732
**At the follow-up**	25.2	18.5	17.0	24.0	0.000		
**Change**	13.5	11.3	8.8	14.9	0.064		0.274
**Ghrelin**							
**At the baseline**	674.8	692.0	672.4	659.4	0.900		
**At the follow-up**	488.0	525.0	424.0	456.8	0.078		
**Change**	-188.0	-168.1	-248.4	-214.0	0.115		
**Adiponectin**							
**At the baseline**	16.4	14.1	14.3	16.0	0.003	b**	0.363
**At the follow-up**	16.5	13.3	14.0	14.7	0.002		
**Change**	0.1	-0.85	-0.3	-1.6	0.261		0.337
**Resistin**							
**At the baseline**	7.7	7.0	7.2	8.2	0.047		0.118
**At the follow-up**	10.9	10.1	11.5	10.8	0.235		
**Change**	2.6	2.7	4.2	3.3	0.020	b*	0.402

Data for continuous variables tested with ANOVA. P-values under 0.05 were considered statistically significant. For post hoc analyses

*P<0.05

**P<0.01

***P<0.001, analyzed using ANOVA Tukey statistical test (95% confidence interval) for differences between groups a) 1 vs 2; b) 1 vs 3 c) 1 vs 4; d) 2 vs 3; e) 2 vs 4; f) 3 vs 4.

#### Telomeres

During the follow-up, the length of telomeres (Table 3) seemed to decrease the most in the “current” and the least in the “never” group (p < 0.001). With post hoc analysis we noticed a statistically significant difference between “never” and all other groups (p-values: from < 0.001 to 0.014). After adjustments for baseline telomere length, smoking pack years, age and sex the statistically significant difference disappeared. It seemed that the difference in telomere shortening was caused by the imbalance of males and females in the groups due telomeres shortened more in males than females. We analyzed also male and female groups separately, but there were no statistically significant differences between groups.

#### Smoking pack years and alcohol consumption

Naturally “never” smokers had smoked the least at the baseline and as well they smoked the least during follow-up (Table 1). “Current” had smoked on average the most at the baseline and they as well smoked the most during follow-up time. However, “quit” group smoking habits seemed similar to “current” group. Their pack years were almost the same at the baseline and they smoked almost as many pack years during the follow-up time. When analyzed sexes separately we noticed that “quit” males smoked almost twice as many pack years compared to “quit” females (males 13.2 pack years and females 6.4 (p <0.001). And “ex-smoker” males had smoked almost 5 times more pack years before quitting than “ex-smoker” females. On the other hand, “current” females smoked 23% more pack years than “current” males during the follow-up time (males 10.7 pack years and females 13.2 (p <0.001)).

Alcohol consumption (g/week) (Table 1) during the follow-up seemed to decrease in the male groups whereas in the female groups, alcohol consumptions remained almost the same. However, the differences in reduction were not statistically significant when comparing the sexes separately, but it seems that males tended to reduce their alcohol consumption more. Among females, only the “current” (4.0g/wk) and “quit” (3.2 g/wk) groups tended to reduce their alcohol consumption, but the reduction was not statistically significant (p = 0.878). Among males, all groups seemed to reduce their alcohol consumption (“never” 18.7 g/wk, “current” 42.3 g/wk, “ex-smoker” 25.1 g/wk and “quit 35.2 g/wk), but the differences were not statistically significant (p = 0.561). Even though the alcohol consumption of males decreased during the study period, the overall consumption of alcohol among the studied males was still greater than among females.

#### Total mortality

Short-term mortality (between the years 2013 and 2014 and by the end of year 2020) was assessed using Cox proportional hazard survival model analysis. The “current” group (HR 2.43; CI 95% 1.10–5.39) but not “ex-smoker” (HR 1.02; CI 95% 0,56–1.86) or “quit” groups (HR 1,43; CI 95% 0.73–2.80) was an independent predictor of total mortality when age, sex and changes (baseline to year 2014) of BMI during follow-up were added to the model (Table 5). The addition of baseline alcohol consumption to the model did not change the results essentially. The mortality rate was also increased in longer follow-up (from the beginning of the follow-up (1993–1994) to the end of the year 2020) in the “current” group.

**Table 5:** Hazard ratios (HR) for total mortality (between 2013 and 2014 and the end of the year 2020) according to smoking status groups (n = 570).

	HR (95% CI)
**Smoking status group**	
**Never**	1
**Current**	2.43 (1.09; 5.38)
**Ex-smoker**	1.00 (0.55; 1.81)
**Quit**	1.42 (0.72; 2.80)
**Age**	1.14 (1.09; 1.19)
**Sex**	1.93 (1.16; 3.19)
**Change of BMI (baseline year 2014)**	0.95 (0.88; 1.03)

Cox regression models representing HR with 95% confidence intervals (CIs) for total mortality. “Never” group = reference group.

## Discussion

The results of our long-term follow-up study show that, as expected, the “quit” group gained the most weight whereas “ex-smokers” gained the least. Systolic blood pressure measured in office conditions decreased the least during the follow-up time among”quit” subjects and the most among “ex-smokers”. When sexes were considered separately, this finding was observed among males. Due to changes in the systolic blood pressure, pulse pressure measured in office conditions also increased the most among “quit” males, and the difference was the most significant compared to “ex-smoker” males. Even though a similar tendency was visible in the ambulatory SBP and PP, the findings were not statistically significant.

### Weight

Previous studies, in accordance with ours, have shown that smoking cessation is related to weight gain [5–7, 11]. In a previous long-term follow-up study, more weight gain occurred in those who quit smoking compared to those who continued smoking or had never smoked [5]. Even though we noticed a significant weight increase in those who “quit”, we did not notice any significant increase in the incidence of diabetes or metabolic syndrome. A recent study also observed that even though smoking cessation was associated with substantial weight gain, it did not increase the risk of chronic diseases and did not attenuate the mortality benefit of cessation [6]. Another study noticed that recent smoking cessation-related weight gain increases the risk of type 2 diabetes, but the risk decreased to the same level with non-smokers over time [8]. Nevertheless, smoking cessation-associated weight gain does not seem to reduce the benefits in the risk on cardiovascular events [10]. A study by Liu G et al. 2020 [35] suggests that smoking cessation without weight gain is a significant factor reducing cardiovascular events and mortality. In an earlier study, smoking cessation along with weight gain reduced less cardiovascular events than without weight gaining. However, the mortality rate remained the same after smoking cessation regardless of whether there was weight gain or not [31]. In our study, the increased short-term mortality observed among persistent smokers was no longer seen among the “quit” group. The benefits of smoking cessation outweigh the chronic disease risks related to weight gain [6].

### Blood pressure

In this study, SBP decreased the most or increased the least in the “current” group, especially among “current” males, and both SBP and DBP decreased the most in the “current” group. After follow-up, subjects belonging to the “current” group had the lowest SBP and DBP. On the other hand, SBP and DBP decreased the least or increased the most in those who “quit”, and after the follow-up, those who “quit” had the highest SBP and DBP. This supports the findings of previous studies that smokers have lower blood pressure than non-smokers and those who quit smoking [11]. Smokers also tend to use less antihypertensive drugs [18]. This lower trend in SBP and DBP is seen in both female and male smokers [13].

In this study, we noticed that pulse pressure increased in all groups, the most in the “quit” and the least in the “current” group. After follow-up, PP was the highest in the “quit” and the lowest in the “current” group. This is in line with the findings of previous studies. It has been seen that smoking cessation increases pulse pressure compared to those who continue smoking [15] and tobacco smoking has a dose-dependent effect on lowering the pulse pressure [17].

Several studies have shown that smoking cessation is associated with increased blood pressure and increased risk of HTN [11, 14, 17]. Blood pressure increased especially in males [13]. Even though in the present study there was significant weight increase in the “quit” subjects and their office SBP and DBP decreased significantly least and their ambulatory SBP increased the most, we did not notice a significant increase in their usage of antihypertensive drugs, the incidence of HTN, or total short-term mortality. Previous studies have also shown that differences in blood pressure levels are significant between “quit” and “current” [11, 13, 14, 17]. However, in this study we observed the most significant differences in blood pressure values after adjustments between those who “quit” and “ex-smokers”.

Alcohol usage affects blood pressure by increasing it, the effect being more significant in males and especially in smokers [13, 36]. It has been shown that reducing the use of alcohol decreases blood pressure levels dose-dependently, and blood pressure decreased the most in those who used 6 or more doses daily and reduced their alcohol intake more than 50% [37]. This is in accordance with our findings. Subjects belonging to the “current” group used alcohol the most and they also reduced their alcohol consumption the most. This reduction in alcohol consumption by “current” subjects may also have contributed to the fact that their blood pressure levels decreased the most, even though these findings were not statistically significant when compared to other groups, partly due to the small number of N in the “current” group.

### Telomere length

Several previous studies show that tobacco smoking promotes shortening of telomeres dose-dependently, and the shortening is associated with increased oxidative stress and chronic inflammation. [21, 23–27] Telomeres also shorten steadily with age [24]. In this study, subjects in the “never” group were the oldest while those in the “current” group were the youngest. This difference in subjects’ age explains why the telomeres were longest in the “current” group at baseline. Females have relatively longer telomeres than males [23] and because females were overrepresented in the “never” group, this may also partly explain why telomeres in the “never” group shortened the least. Smoking and metabolic syndrome-related factors cause increased shortening of telomeres [38]. However, some studies have noticed that high BMI reduced the effect of tobacco smoking on telomere shortening [39]. Even though telomeres seemed to shorten the most in “current” group and the least in “never” group the imbalance of males and females in these groups mostly explains this visible difference. The difference in telomere shortening between groups was not statistically significant after adjustments.

Both smoking and alcohol usage cause lack of antioxidative defense, which contributes to telomere shortening [27]. Telomere shortening has been observed especially in binge drinking [40]. However, most studies have shown that moderate alcohol use does not have affect telomere length [40, 41]. A 10-year follow-up study did not observe any effect on telomere length by alcohol usage [41]. However, a Finnish cohort study found that moderate alcohol use in middle age had an impact on telomere shortening later on [42]. In our study, “current” males consumed the most alcohol, and this may partly explain why their telomeres shortened the most. But as mentioned earlier we didn’t notice statistically significant differences in telomere lengths between groups.

### Confounding factors

We noticed that, fortunately, the majority of those who smoked at baseline quit during the follow-up. That left us with quite a small number of “current” subjects after the follow-up (only 45), and this prevalence issue may be among the reasons why some variables did not become statistically significant.

We also noticed that sex was a significant confounding factor in our study. For example, it had a major impact on telomere length, office PP and night-time ambulatory diastolic blood pressure changes. In addition, when dividing the study population into groups by sex, the number of “current” group subjects was too low to make relevant comparisons. Along with physiological differences we also noticed some habitual differences between females and males, which may partly explain why sex was a major confounding factor. Especially smoking habits and alcohol consumptions differed between the sexes. When studying the differences in these smoking habits the question arises of how the number of smoking pack years affects the variables in question. Males who quit smoking during the follow-up smoked almost twice as many pack years compared to females who quit during the follow-up. An earlier study found that after smoking cessation, blood pressure levels were higher after one year compared to those who continued smoking [16]. This may indicate that males who quit during the follow-up had quit more recently and the blood pressure-increasing effect was stronger.

## In conclusion

In the “quit” group, office systolic blood pressure decreased the least and pulse pressure increased the most. The differences were most significant compared to “ex-smokers” and were noticeable in the whole study group, but especially in males. Even though there was a noticeable increase in weight and waist circumference in the “quit” group, there was no significant increase in the incidence of obesity-related diseases, such as metabolic syndrome, hypertension and diabetes. Unlike in the “current” group, short-term mortality among the “quit” subjects was no longer increased compared to the “never” or “ex-smoker” groups. Therefore, the benefits of smoking cessation outweigh the disadvantages in the long term.
