# Correction: Smoking cessation and obesity-related morbidities and mortality in a 20-year follow-up study

**DOI:** 10.1371/journal.pone.0306217

**Published:** 2024-06-21

**Authors:** Asla Suutari-Jääskö, Antti Ylitalo, Justiina Ronkainen, Heikki Huikuri, Y. Antero Kesäniemi, Olavi H. Ukkola

The third author’s name is spelled incorrectly. The correct name is: Justiina Ronkainen. The correct citation is: Suutari-Jääskö A, Ylitalo A, Ronkainen J, Huikuri H, Kesäniemi YA, Ukkola OH (2022) Smoking cessation and obesity-related morbidities and mortality in a 20-year follow-up study. PLoS ONE 17(12): e0279443. https://doi.org/10.1371/journal.pone.0279443.
